# Tension Orbital Emphysema Following Blunt Trauma: A Case of Orbital Compartment Syndrome

**DOI:** 10.7759/cureus.97421

**Published:** 2025-11-21

**Authors:** Chang Feng Chew, Vindhya Prem-Kumar

**Affiliations:** 1 Department of Ophthalmology, Hospital Raja Permaisuri Bainun, Ipoh, MYS

**Keywords:** blunt trauma, emergency, lateral canthotomy, orbital compartment syndrome, orbital emphysema, orbital wall fracture, retrobulbar hemorrhage, tension orbital emphysema, tension pneumo-orbit

## Abstract

Orbital emphysema commonly occurs following traumatic orbital fractures, which usually resolve spontaneously. However, in rare cases, it can progress to tension orbital emphysema, leading to orbital compartment syndrome (OCS), a potentially blinding condition if not treated promptly. We report a 47-year-old man who developed OCS one day after sustaining blunt trauma to his right cheek. Initial examination revealed periorbital hematoma, emphysema, chemosis, and a raised intraocular pressure (IOP) of 28 mmHg without proptosis. Computed tomography showed a right orbital floor blowout fracture with orbital emphysema. His condition worsened the following day with a new onset of proptosis, total ophthalmoplegia, and an IOP of 48 mmHg. Emergency right lateral canthotomy and systemic acetazolamide reduced IOP to 28 mmHg, with full recovery after five days with 6/9 visual acuity, no relative afferent pupillary defect, resolved chemosis, normalized IOP and full range of extraocular movement. He remained stable for two weeks, after which he was lost to follow-up. This case highlights the importance of early recognition and prompt management of tension orbital emphysema to prevent permanent visual loss.

## Introduction

Orbital emphysema occurs when air from the paranasal sinuses is trapped within the soft tissues of the orbit [1,2]. In most cases, orbital emphysema is treated conservatively as it is a benign condition and resolves after a few days or weeks [1,2]. On rare occasions, there is a buildup of trapped air in the orbit space, causing tension orbital emphysema, resulting in orbital compartment syndrome (OCS) [1,2]. Blowout fractures of the orbital wall following facial trauma are well-documented, but the development of orbital emphysema and its sequelae in the form of increased intraocular pressure (IOP) is a rare occurrence [1-5]. We report a rare case of OCS developing secondary to orbital emphysema following an orbital floor blowout fracture, which is an uncommon and vision-threatening complication of a typically benign condition.

## Case presentation

A 47-year-old healthy gentleman was referred for eye assessment following a motorcycle accident one day prior, during which he sustained blunt force trauma to his right cheek. He complained of right periorbital swelling with a "bubbly appearance". Upon examination, he exhibited right periorbital hematoma with crepitation and subconjunctival emphysema with chemosis, giving a "bubbly appearance". His presenting visual acuity (VA) was 6/9 bilaterally with no relative afferent pupillary defect (RAPD) or proptosis. There was a slight limitation of his right eye abduction, and his intraocular pressure (IOP) was 28 mmHg measured with a Goldmann applanation tonometer (GAT). The posterior segment of the right eye was unremarkable, and no abnormalities were detected in his left eye. A computed tomography (CT) scan of the face showed a right orbital floor blowout fracture with orbital emphysema in the extraconal space. The patient was started on topical antiglaucoma and chloramphenicol eyedrops.

**Figure 1:**
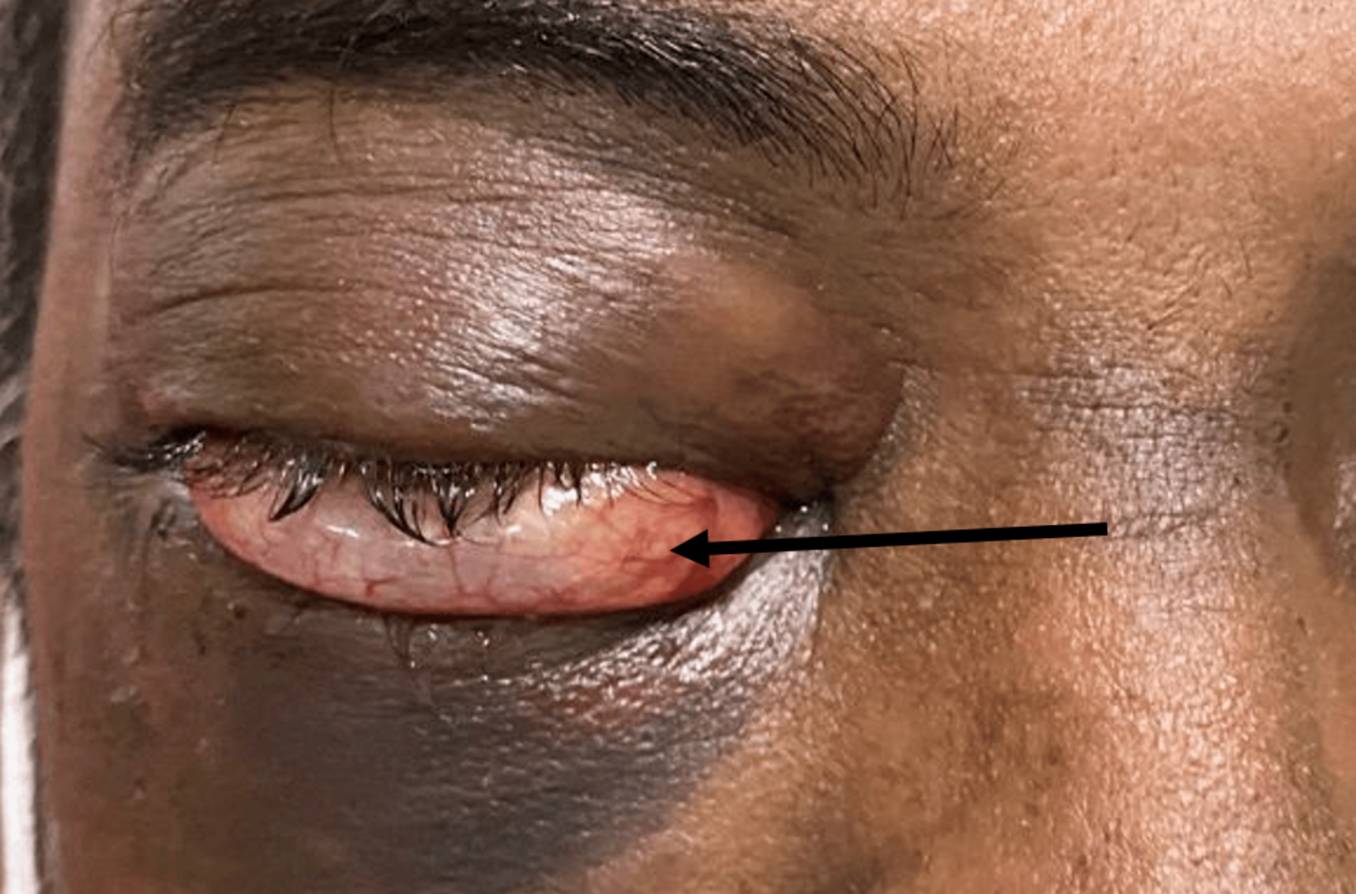
A clinical photograph showing right eye periorbital hematoma and inferior prolapse of conjunctiva with worsening chemosis (black arrow)

His right eye symptoms worsened the following day with new-onset proptosis, a tense globe, and worsening chemosis (Figure 1) with restricted extra-ocular movements in all directions. VA remained 6/9 with intact optic nerve function. IOP, however, was significantly elevated to 48 mmHg on GAT. An emergency right lateral canthotomy without cantholysis was performed, and systemic acetazolamide was administered promptly, which relieved his IOP to 28 mmHg. Cantholysis was not performed as the globe was noticeably less tense following canthotomy alone, indicating adequate decompression. Optic nerve function remained intact, and there was no further rise in IOP thereafter.

**Figure 2:**
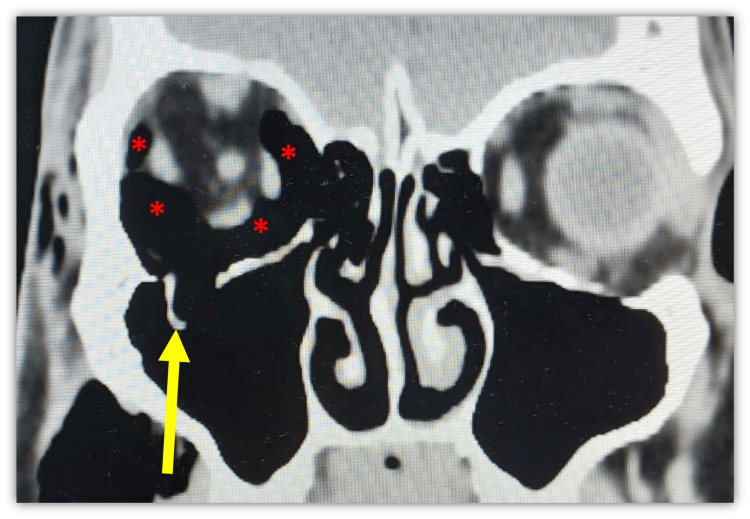
Coronal CT orbit showing right extra-conal orbital emphysema (red asterisks) with a right inferior blowout fracture (yellow arrow) CT: Computed tomography

**Figure 3:**
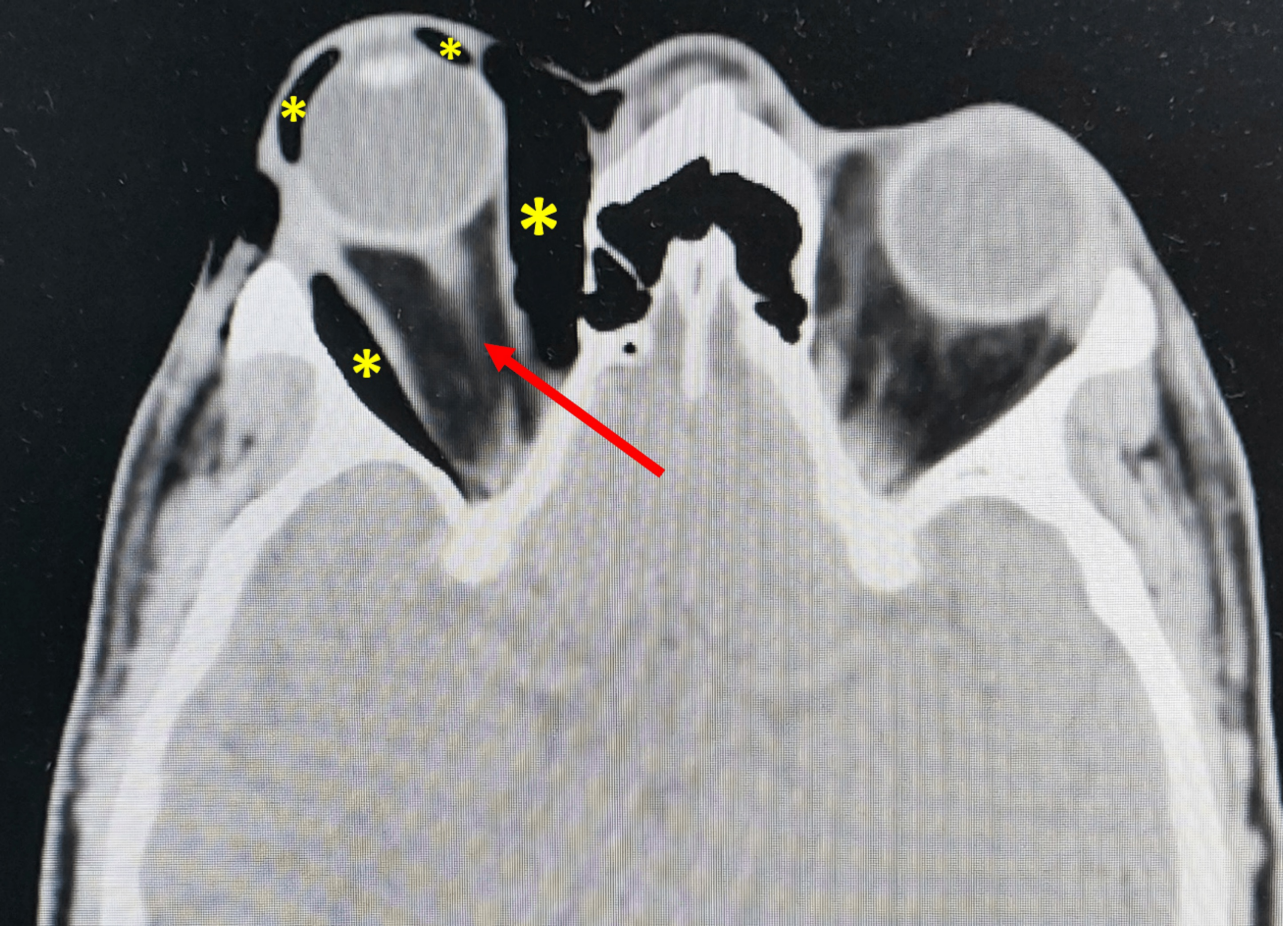
Axial CT orbit showing right globe proptosis with orbital emphysema (yellow asterisks) and stretching of the optic nerve (red arrow) CT: Computed tomography

**Figure 4:**
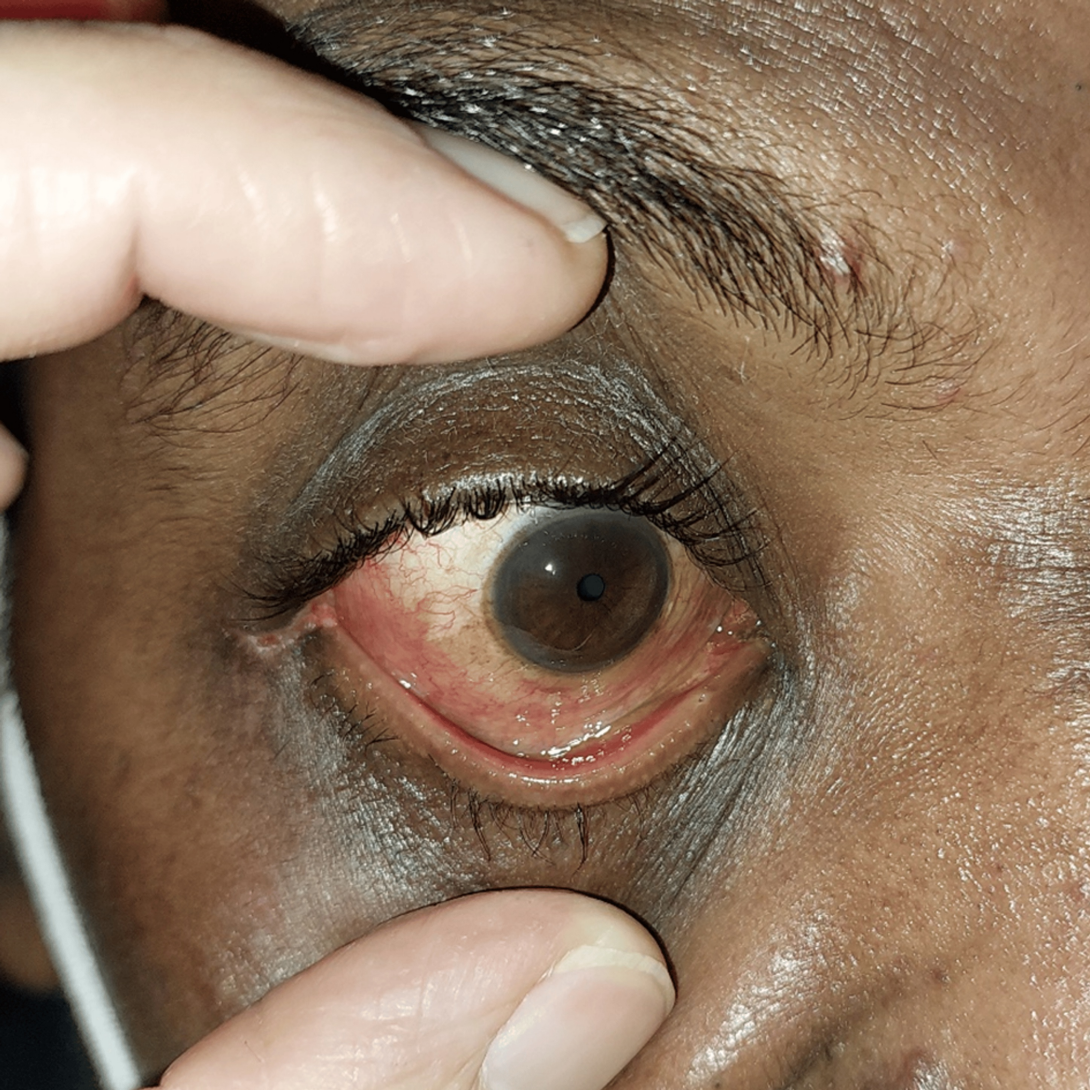
A clinical photograph of the right eye showing resolution of the chemosis and proptosis

A repeated urgent CT orbit showed similar orbital emphysema with new findings of proptosis and stretching of the optic nerve (Figure 2, Figure 3). There was no evidence of orbital cellulitis, cavernous sinus thrombosis or carotico-cavernous fistula. He received inpatient care with systemic and topical antiglaucoma. His symptoms resolved completely, and ocular condition normalized after five days with normal IOP, full range of extraocular movements, and VA of 6/9 (Figure 4). At two weeks post-discharge, his ocular status remained stable and unremarkable, with no recurrence or delayed complications, after which he was lost to follow-up.

## Discussion

The average adult orbit has a volume of approximately 30 mL. It forms a confined, cone-shaped space with limited compliance due to the restricted elasticity of the septum and tarsal plates [3]. This anatomical structure increases the risk of OCS when intraorbital volume rises. Retrobulbar hemorrhage is the leading cause of OCS, often resulting from trauma or iatrogenic causes [3,4]. Additional etiologies include Valsalva-related hemorrhage, vascular malformations, and periocular or facial chemical burns that cause fluid egression. Traumatic asphyxiation is also a cause [3,4]. Bleeding disorders such as hemophilia, leukemia, and von Willebrand disease have been implicated in the development of OCS [4]. Infective causes, like fulminant orbital cellulitis with or without an orbital abscess, have also been reported to induce OCS [3,4]. In rare cases, orbital emphysema may result in OCS after traumatic orbitofacial fractures or surgical procedures involving the orbit, eyelid, lacrimal system, or sinuses [3-5]. Orbital emphysema occurs when air becomes trapped within the orbital soft tissues. It is a recognized complication of orbital fractures and is typically managed conservatively [1,2,4]. This condition is often associated with nose-blowing or sneezing after orbital wall fractures. These actions can force nasal air into the intraorbital soft tissues through defects between the sinus and the orbit [1-3,5]. Rarely, the orbital soft tissues may block the bony defect, forming a one-way check valve. This allows air in but retains it, leading to a progressive rise in orbital pressure (also known as tension orbital emphysema) and eventually to OCS [1,2,5].

Similar cases of OCS secondary to orbital emphysema have been reported, which are very rare. Ashman et al. (2020) [1] and Shrivastava et al. (2022) [5] described patients who developed OCS following minor trauma, both requiring urgent lateral canthotomy with cantholysis. Lin et al. (2016) [2] reported a patient who was successfully treated with needle decompression without canthotomy, demonstrating that various decompression techniques can be effective depending on clinical severity. Similarly, Sheele and Lang (2016) [4] reported a case managed with lateral canthotomy alone, in which the IOP decreased from 54 mmHg to 28 mmHg following the procedure. In our case, there was no evidence of optic neuropathy, as the patient maintained good VA and no RAPD. Hence, a staged approach was undertaken, beginning with lateral canthotomy, with the option to proceed to cantholysis if decompression proved inadequate. Lateral canthotomy alone effectively reduced the intraocular pressure and improved globe tension, leading to full recovery. These comparisons emphasize that early recognition and prompt, tailored decompression, whether by canthotomy, cantholysis, or needle decompression, are crucial in preventing irreversible visual loss.

OCS is an ocular emergency and a clinical diagnosis. Once suspected, treatment should not be delayed due to its potential blinding complications [1-6]. This rapid-onset, elevated orbital tension may compress the optic nerve and/or the central retinal vessels. This can result in hypoperfusion and eventually visual loss [1-4]. It has been reported that after three hours, ischemia occurs, leading to permanent visual compromise [4]. Marked eyelid swelling, proptosis, retropulsion resistance on digital ocular palpation, diffuse subconjunctival hemorrhage, chemosis, visual loss or diplopia, presence of RAPD, partial or complete ophthalmoplegia, and increased IOP are signs suggestive of tension orbital emphysema [3,4]. A CT is not mandatory, but it is useful for confirming the diagnosis. It may show straightening of the optic nerve or “globe tenting” [3]. In most cases, entrapped air is localized superiorly and medially in the orbital extraconal space. As a result, a crescent-shaped radiolucency can be seen on radiographs. This "black eyebrow sign" is highly suggestive of orbital emphysema [5].

Orbital emphysema is commonly benign and self-limiting. It resolves in a few days to weeks [1,2,4,5]. However, urgent surgical intervention is indicated when OCS has occurred. Procedures such as lateral canthotomy with or without cantholysis, needle decompression, or surgical orbital decompression by an open or endoscopic approach have all been mentioned in the literature [1-6]. In this case report, the patient demonstrated most of the clinical signs and CT findings previously described. Therefore, early recognition and prompt treatment in the presence of emphysema with raised IOP remain crucial to prevent unwanted blinding complications.

## Conclusions

In conclusion, we reported a rare, rapidly progressing case of orbital emphysema with vision-threatening tension orbital emphysema following an orbital floor blowout fracture that required emergency lateral canthotomy to lower IOP. This case underscores the critical need for instant recognition. Clinicians must urgently suspect OCS and perform prompt decompression in any case of emphysema and elevated IOP to avert irreversible vision loss.

## References

[1] Ashman A, Norris JH, Chaidas K (2020). Tension pneumo-orbit secondary to minor blunt force trauma. Eur Ann Otorhinolaryngol Head Neck Dis.

[2] Lin CY, Tsai CC, Kao SC, Kau HC, Lee FL (2016). Needle decompression in a patient with vision-threatening orbital emphysema. Taiwan J Ophthalmol.

[3] McCallum E, Keren S, Lapira M, Norris JH (2019). Orbital compartment syndrome: an update with review of the literature. Clin Ophthalmol.

[4] Sheele J, Lang J (2016). A rare case of traumatic tension pneumo-orbitum. Emerg Med.

[5] Shrivastava AK, Rao S, Nayak S, Rao S, Anto M (2022). Orbital and intracranial emphysema causing orbital compartment syndrome: a rare case report and literature review. Indian J Otolaryngol Head Neck Surg.

[6] Al-Shammari L, Majithia A, Adams A, Chatrath P (2008). Tension pneumo-orbit treated by endoscopic, endonasal decompression: case report and literature review. J Laryngol Otol.

